# Case of Polydipsia

**Published:** 1816-03

**Authors:** John Ware


*For the London Medical and Physical Journal.*

### *Case of Polydipsia*; by John Ware, Esq.

THE subject of the following account is a young man, named James Webb, now living in the town of Hingham, in this state. Having been informed of his singular case, I went in company with Mr. Norton, Librarian of Harvard University, to ascertain its nature and the truth of the circumstances related concerning him. During our visit, the following facts were collected from his own account of himself, which were confirmed by the testimony of the persons with whom he now lives, and, as will be presently stated more particularly, by that of others with whom he has formerly lived.

He was twenty years of age some time in the month of

 October,

October, 1814. His appearance is that of firm health; his complexion is dark and ruddy; he is short, thick, rather sturdy, and, except his preternatural thirst, has never been afflicted with disease. At present, the quantity which he habitually drinks in twenty-four hours, amounts to three pailsful, or six gallons. This is necessary, not only to prevent the sensation of a tormenting thirst, but to preserve him in his ordinary state of health; for, when he abstains from his usual allowance, his head is affected and he becomes dizzy, weak, and sick; or, as he himself expressed it:—"When I don't drink, it gets into my head." He finds himself obliged to drink at intervals of about an hour and an half, or two hours, one or two quarts at a time. At night he places a pailful of water at his bedside, the whole of which he requires before morning, waking whenever it becomes necessary. He has sometimes taken to the amount of a gallon at once, without experiencing any bad effects. He drank two quarts in our presence, having taken one but fifteen minutes before. He swallows with great eagerness and the appearance of satisfaction, drinking a quart in the time that a common person would a quarter of that quantity. He uses water directly from the well, even in the depth of winter, and avoids mixing any thing with it, especially any kind of spirituous liquors, which he dislikes. Its coldness causes no inconvenience, except occasionally a slight chill. He has no recollection of the time when this habit commenced, but has been told by his parents that it was in infancy and soon after birth. The quantity which he now drinks, does not, he thinks, differ materially from that which he drank at nine or ten years old. He has several times endeavoured to break off the habit, but has always suffered from the attempt in the manner above mentioned. His appetite for food is not remarkable: the persons with whom he lives merely observing, that he was a hearty eater. His meat and drink at meals are like those of the persons with whom he lives. His pulse during our visit, was full, strong, and remarkably infrequent, not exceeding, at any time, fifty-six pulsations in a minute, and being sometimes so few as forty-five. It varied as follows:—

Some time after drinking, fifty-six.

Fifteen minutes after drinking, and just before drinking again, fifty.

Immediately after drinking two quarts, forty-five.

The temperature of the atmosphere has no influence on his thirst, since he requires the same quantity of water in the warmest, as in the coldest weather. He had an uncle who was formerly affected in the same way, although not to an equal equal degree. He served in the army during the revolutionary war, and was said to have died in consequence of being in a situation where water was not to be obtained.

As would be supposed, these extraordinary quantities of fluid are wholly carried off by the kidnies, and affect the secretion of no other part. His urine, he thinks, equal in quantity to the whole of the water which he drinks. The qualities of the secreted fluid, I had no opportunity of examining. He perspires very little, and his fæces are of the usual healthy consistence.

This account is confirmed by Messrs. Wilder and Hersey, two persons with whom he formerly resided. With Mr. Wilder, he lived some time at about the age of nine or ten years, and his relation agrees with that of Webb in every particular, especially as to the quantity of water drank during twenty-four hours.

With Mr. Hersey, Webb lived from fourteen to eighteen years of age. He thinks that the quantity increased while he was with him, and feels confident, that during the latter part of the time he drank as much as four pailsful, or eight gallons, daily, and has known him to drink a gallon at a time. He never knew him suffer from want of water but once, when away from home where none was to be obtained; he looked pale and said he could not live ten minutes longer, but was immediately relieved by drinking. He used to shudder for ten or fifteen minutes after drinking, so that his teeth would chatter. His health and appetite were good. He used no spirit. Mr. Hersey says, that he has understood from the person with whom he lives now, that the quantity has diminished since Webb has been with him.

In the second and third volumes of A Collection of Medical Facts, published in London in 1792, are accounts of three cases similar to that above detailed, of which, for the sake of comparison, it may be worth while to subjoin a short notice. The first was that of Catharine Bousergent, a French woman, forty years of age. The disease had existed from infancy. When single she drank three pailsful daily; but after marriage, when pregnant, only two. The quantity was stated by others to be sometimes four pailsful.

The second case was that of a man, in England, aged fifty-one. The disease had existed twenty-three years, and came on after a long continued fever and ague. The quantity he drank amounted to sixteen or seventeen quarts daily.

The third subject was a boy of five years; his pulse from eighty to eighty-five. His daily quantity amounted to about ten quarts.—*New-England Journal.*

